# A Chromosome-Level Genome Assembly and Evolution Analysis of *Andrena camellia* (Hymenoptera: Andrenidae)

**DOI:** 10.1093/gbe/evad080

**Published:** 2023-05-12

**Authors:** Kaixuan Zhao, Arong Luo, Qingsong Zhou, Wei Wei, Wenping Liu, Chaodong Zhu, Zeqing Niu, Zeyang Zhou, Dunyuan Huang

**Affiliations:** Key Laboratory of Conservation and Utilization of Pollination Insects of the Upper Reaches of the Yangtze River (Co-construction by Ministry and Province), Ministry of Agriculture and Rural Affairs, P. R. China, Chongqing, China; Chongqing Key Laboratory of Vector Insects, Chongqing Normal University, Chongqing, China; Key Laboratory of the Zoological Systematics and Evolution, Institute of Zoology, Chinese Academy of Sciences, Beijing, China; College of Biological Sciences/International College, University of Chinese Academy of Sciences, Beijing, China; Key Laboratory of the Zoological Systematics and Evolution, Institute of Zoology, Chinese Academy of Sciences, Beijing, China; Key Laboratory of Conservation and Utilization of Pollination Insects of the Upper Reaches of the Yangtze River (Co-construction by Ministry and Province), Ministry of Agriculture and Rural Affairs, P. R. China, Chongqing, China; Chongqing Key Laboratory of Vector Insects, Chongqing Normal University, Chongqing, China; Key Laboratory of Conservation and Utilization of Pollination Insects of the Upper Reaches of the Yangtze River (Co-construction by Ministry and Province), Ministry of Agriculture and Rural Affairs, P. R. China, Chongqing, China; Chongqing Key Laboratory of Vector Insects, Chongqing Normal University, Chongqing, China; Key Laboratory of the Zoological Systematics and Evolution, Institute of Zoology, Chinese Academy of Sciences, Beijing, China; College of Biological Sciences/International College, University of Chinese Academy of Sciences, Beijing, China; State Key Laboratory of Integrated Pest Management, Chinese Academy of Sciences, Institute of Zoology, Beijing, China; Key Laboratory of the Zoological Systematics and Evolution, Institute of Zoology, Chinese Academy of Sciences, Beijing, China; Key Laboratory of Conservation and Utilization of Pollination Insects of the Upper Reaches of the Yangtze River (Co-construction by Ministry and Province), Ministry of Agriculture and Rural Affairs, P. R. China, Chongqing, China; Chongqing Key Laboratory of Vector Insects, Chongqing Normal University, Chongqing, China; Key Laboratory of Conservation and Utilization of Pollination Insects of the Upper Reaches of the Yangtze River (Co-construction by Ministry and Province), Ministry of Agriculture and Rural Affairs, P. R. China, Chongqing, China; Chongqing Key Laboratory of Vector Insects, Chongqing Normal University, Chongqing, China

**Keywords:** *Andrena camellia*, chromosome-level assembly, comparative genome, genome evolution

## Abstract

*Andrena camellia*, an effective pollinator of the economically significant crop *Camellia oleifera*, can withstand the toxic pollen of *C. oleifera*, making *An. camellia* crucial for resource conservation and cultivation of *C. oleifera.* In this study, the whole genome of *An. camellia* was sequenced on the Oxford Nanopore platform. The assembled genome size was 340.73 Mb including 50 scaffolds (N50 = 47.435 Mb) and 131 contigs (N50 = 17.2 Mb). A total of 11,258 protein-coding genes were annotated; in addition, 1,104 noncoding RNAs were identified. Further analysis shows that some chromosomes of *An. camellia* have a high level of synteny with those of *Apis mellifera*, *Osmia bicornis*, and *Andrena minutula*. Thus, our reported genome of *An. camellia* serves as a valuable resource for studying species evolution, behavioral biology, and adaption to toxic pollen of *C. oleifera*.

**Significance**

As the main pollinators of *Camellia oleifera*, *Andrena camellia* (Andrenidae) is widely distributed in the *C. oleifera* plantation of China and characterized by underground nesting. In this study, we sequenced and assembled the genome of *An. camellia* and obtained a high-quality genome. The results of our study are valuable for further studies of this species’ evolution and behavioral biology.

## Introduction

Currently, in the wasp and bee superfamily Apoidea (Hymenoptera), over 20,000 species of bee have been identified and are widely distributed around the world (Danforth et al. 2013; Ascher and Pickering 2020). The family Andrenidae is a well-established clade of the bee family, containing more than 3,000 species (Ascher and Pickering 2020). Approximately 1,550 species have been described in the *Andrena* genus (Ascher and Pickering 2020). Although the taxonomy and phylogeny of this genus have been well studied, molecular phylogeny and evolution have rarely been addressed. However, *Andrena minutula* has been sequenced and assembled in 358 sequence scaffolds with a total length of 380 Mb and a scaffold N50 of 50.2 Mb, and 92.19% assembly sequence was assigned to seven chromosomes (Falk and Blomfield-Smith 2022).

*Andrena camellia* is a typical solitary bee, whose foraging behavior can improve the fruit setting rate of *Camellia oleifera*. Therefore, it was considered as an effective pollinator (Huang et al. 2021; Li et al. 2021). However, studies have shown that the pollen of *C. oleifera* is toxic to many bee species, which prevents bee colonies from feeding on it (Su et al. 2011). Therefore, we are interested in the possible reasons why *C. oleifera* pollen is not poisonous to *An. camellia*. To investigate these issues and generate a needed community resource, we sequenced the entire genome of *An. camellia* and annotated noncoding RNAs, genomic elements, protein-coding genes (PCGs), and repeat sequences. In addition, an evolution analysis of gene families of several important bee lineages was carried out. In conclusion, this study not only provides important data for an in-depth understanding of the evolutionary history and behavioral biology of *An. camellia*, but also helps to elucidate its resistance to toxic pollens.

## Results and Discussion

### Genome Sequencing and Assembly

A total of 38.32 Gb second-generation-sequencing clean reads (N50 = 21.01 kb) and 33.28 Gb nanopore clean data (N50 = 6.15 kb) were obtained. In addition, 40.99 Gb data were obtained on the Illumina NovaSeq 6000 platform, including 10.02 Gb transcriptome reads and 30.97 Gb Hi-C reads. The predicted genome size is 332.84 Mb, with low heterozygosity (supplementary table S1 and fig. S1, Supplementary Material online).

**Fig. 1.**
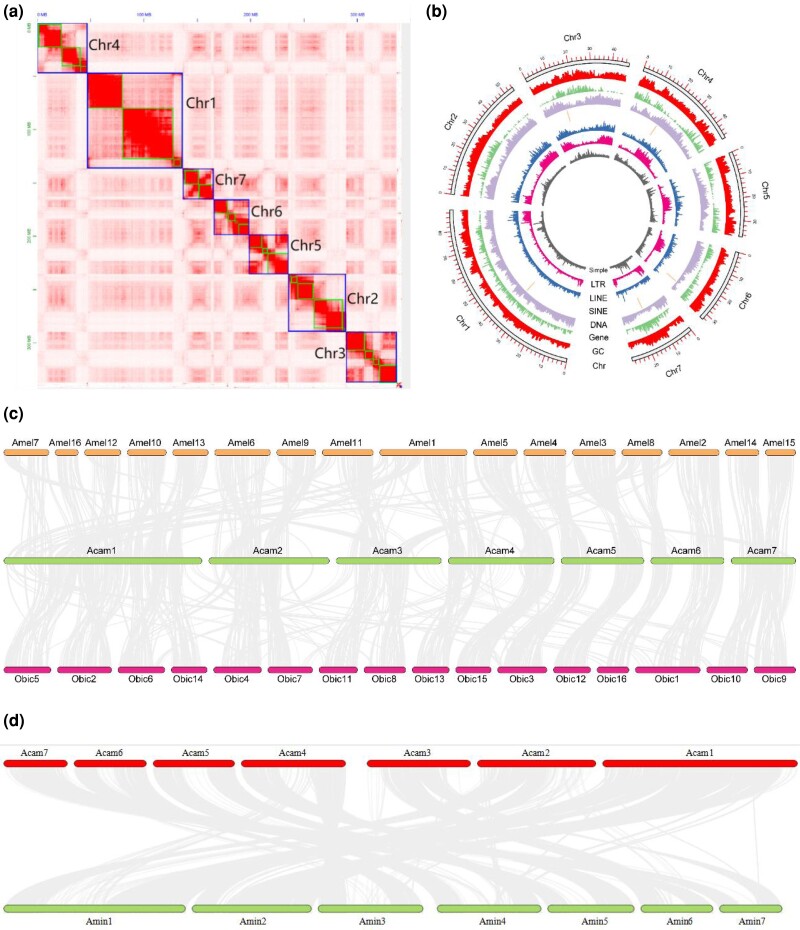
Assessment of the *Andrena camellia* genome (*a*) Hi-C heat map of *An. camellia*, the large boxes corresponding to Chr1-7 indicate the super scaffolds, and contigs are shown as small boxes within. (*b*) *Andrena camellia* genome characterization map showing the following genomic characteristics: SINE/LINE/LTR retrotransposons, DNA transposons (DNA), chromosome length (Chr), GC content (GC), density of PCGs (GENE). (*c*) Chromosome covariance and genome structure. Amel1-16 represents the first to the 16th chromosomes of *Apis mellifera*. Obic1-16 represents the first to 16th chromosomes of *Osmia bicornis*. Acam1-7 represents the first to seventh chromosomes of *Ap. mellifera.* (*d*) Synteny, among *An. camellia* and *An. minutula.* Acam1-7 represents the first to seventh chromosomes of *Ap. mellifera*, and Amin1-7 represents the first to seventh chromosomes of *An. minutula*.

The assembly size after polishing was 341.85 Mb (table 1). After manual corrections, the final assembled genome size of *An. camellia* was 340.73 Mb, including 125 scaffolds (maximum length = 89.225 Mb, N50 = 47.435 Mb) and 50 contigs (maximum length = 47.974 Mb, N50 = 17.2 Mb). There was 42.25% GC content in the genome. The assembled genome size was consistent with the expected. Hi-C scaffolding assigned 98.95% (337.159 Mb) of the genome to 7 pseudo-chromosomes (listed with size): 1) 89.23 Mb, 2) 54.26 Mb, 3) 47.46 Mb, 4) 47.36 Mb, 5) 36.88 Mb, 6) 32.78 Mb, and 7) 29.21 Mb (fig. 1*a*).

**Table 1:** Assembly Statistics of the *Andrena camellia* Genome Across Different Stages of Genome Assembly

Assembly	Total Length (Mb)	Number of Scaffolds/Contigs	Scaffold/Contigs N50 Length (Mb)	Longest Scaffold/Contigs (Mb)	GC (%)	BUSCO (*n* = 1,367) (%)			
						C	D	F	M
NextDenovo	341.628	53/53	19.854/19.845	54.844/54.844	42.22	98.6	0.1	0.6	0.8
NextPolish	341.849	53/53	19.876/19.876	54.9/54.9	42.23	99.2	0.1	0.2	0.6
3D-DNA	341.866	69/185	47.437/17.2	89.225/47.974	42.23	99.1	0.1	0.2	0.7
Final	340.73	50/131	47.435/17.2	89.225/47.974	42.25	99.1	0.1	0.2	0.7
MAKER						98.6	15.1	0.4	1.0

Note.—C, D, F, and M represents complete BUSCO, complete and duplicated BUSCOs, fragmented BUSCOs, and missing BUSCOs, respectively. MAKER is for Integrity Assessment of Predicted Protein-Coding Genes.

The genome integrity was assessed using the insect BUSCO reference database (genes = 1,367). The integrity of the initial assembly increased through to the final version, with a nearly complete single-copy set of BUSCO genes, and low duplication rate (table 1). The mapping rates of the second-generation whole-genome data, RNA-seq and the third-generation nanopore ONT raw sequences were 98.59%, 95.26%, and 98.63%, respectively. These results indicate that our genome assembly had high gene content with minimal duplicate content.

Compared with the genomes available in the family Andrenidae, our genome size is smaller than *Andrena hattorfiana* (428.5 Mb), *Andrena fulva* (461.7 Mb), and *Andrena bucephala* (379.8 Mb); larger than *Andrena haemorrhoa* (330.7 Mb) and *Andrena dorsata* (277.3 Mb); slightly less than *An. minutula* (380.4 Mb), though the contig N50 is much longer than most of these previous assemblies (*An. minutula*: 12.7 Mb; *An. hattorfiana*: 10.2 Mb; *An. haemorrhoa*: 13.2 Mb; *An. bucephala*: 8 Mb; *An. fulva*: 12.5 Mb), only smaller than *An. dorsata* (30.1 Mb) (supplementary table S7, Supplementary Material online).

### Genome Annotation

RepeatMasker analysis showed that there are 508,701 repeats in the whole genome, accounting for 44.65% of the content. Further analysis revealed that the highest percentage of repeats was DNA elements (16.03%), followed by unknown (16.75%), long-terminal repeats (LTRs; 6.79%), long interspersed elements (LINEs; 1.08%), and simple repeats (0.73%; supplementary table S2, Supplementary Material online).

Through the MAKER pipeline, 11,258 PCGs were annotated with an average length of 7,029 bp. The average number of exons per gene is 6.9 and the average length is 360.6 bp, whereas the number of introns per gene is 5.9 and the length is 839.2 bp. In addition, there were 6.7 CDS per gene, with the mean length 259.7 bp. BUSCO analysis identified 1,141 single-copy, 5 fragmented, 14 missing, and 207 duplicated BUSCOs (table 1). The genome contains 10,866 genes encoding 9,342 proteins (82.98%). A total of 8,602 and 4,049 PCGs were assigned to GO and Kyoto Encyclopedia of Genes and Genomes (KEGG) terms through InterProScan and eggNOG-mappers (supplementary table S3, Supplementary Material online and fig. 1*b*). In addition, 1,104 ncRNAs were identified, including 92 micro-RNAs, 448 rRNAs, 48 small nuclear RNAs (snRNA), 171 tRNAs, and 323 others. Among them, snRNAs include 35 spliceosomal RNAs, 3 minor spliceosomal RNAs, 8 C/D box snoRNAs, and 3 haca box snoRNAs (supplementary table S4, Supplementary Material online).

### Gene Family Identification and Chromosomal Synteny

Gene families were identified by OrthoFinder using 125,440 genes from 11 hymenopterans as a reference. A total of 121,326 genes were assigned to 11,665 gene families, of which 4,892 were single-copy orthologs and 1,426 were multicopy orthologs (supplementary table S5, Supplementary Material online). There are 4,892 single-copy orthologs and 1,833 multicopy orthologs in *An. camellia* genome, including 28 genes shared by Apoidea and 104 *An. camellia* specific genes (supplementary table S6 and fig. S2, Supplementary Material online).

Comparative genomic assessment showed a significant correspondence between the chromosomes of *An. camellia*, *Apis mellifera*, and *Osmia bicornis*. For example, chromosome 7 of *An. camellia* had extensive synteny with chromosomes 14 and 15 of *Ap. mellifera*, chromosomes 9 and 10 of *O. bicornis*, chromosome 7 ends and chromosome 8 of *Ap. mellifera* have homologous segments on chromosome 3 of *An. camellia*, the terminal differentiation of *An. camellia* chromosome 6 into *Ap. mellifera* chromosome 8 and *O. bicornis* chromosome 10. In comparison with *An. minutula*, there was a clear one-to-one block between our assembled genome and the *An. minutula* genome, that is, they was high collinearity and the syntenic regions of *An. camellia* chromosome 4 were located on chromosome 1, 3, and 5 of *An. minutula*, which provides evidence for the phylogeny of Apoidea at the chromosomal level (fig. 1*c* and *d*).

## Materials and Methods

### DNA and RNA Sequencing

*Andrena camellia* was collected at the *C. oleifera* base in Shiti Town, Xiushan, Chongqing (28.43N, 109.13E), and the samples were placed in liquid nitrogen and stored at −80 °C before being sent to Beijing Berry Genomics for sequencing. Species identification was performed using universal primers (LCO1490 and HCO2198) for amplification and blast on NCBI (Folmer et al. 1994). A 1D DNA ligation sequencing kit (SQK-LSK109) was used to extract genome DNA for single-molecule sequencing using Oxford nanopore triple sequencing. For nanopore sequencing, an insert with a length of 40 kb was created, whereas in second-generation sequencing based on CTAB method, a 350-bp insert size was created for sequencing at the Beijing Genomics Institute. RNA was extracted and libraries were constructed using TRIzol Reagent and TruSeq RNA v2 kits, respectively; Hi-C library was also constructed for Illumina NovaSeq 6000 sequencing (2 × 150 bp), one specimen each for Nanopore, second-generation, transcriptome, and Hi-C sequencing.

Quality control and trimming of raw data were performed by BBTools v38.82 (Bushnell 2014). Duplicate sequences were moved by clumpify.sh-, whereas bbduk.sh was used for specific quality control, removal of sites with a base quality score below 20 (>Q20), filter sequences below 15 bp in length, trim-off poly-A/G/C tails longer than 10 bp, then compare orthobases using overlapping reads.

We also performed K-mer analysis using GenomeScope v2.0 (Ranallo-Benavidez et al. 2020) to predict genome size, the maximum k-mer coverage cutoff is set to 10,000, the k-mer frequencies are evaluated using khist.sh (BBTools) with the length set to 21 -mer., and the GenomeScope parameter is set to: -k 21 -p 2 -m 10000.

### Genome Assembly

Genome was assembled using NextDenovo V2.5.0 (https://github.com/Nextomics/NextDenovo; read_cutoff = 1k). To move forward, gathering precision grouping rectification was performed utilizing NextPolish v1.4.0 (Hu et al. 2020), and the required genome sequence alignment file bam was generated by Minimap2 v2.4 (Li 2018) by aligning the second/third-generation data to the third-generation assembled genome. The chromosomal level of genome assembly was aided by Hi-C sequencing reads. Juicer V1.6.2 performed the quality control on the Hi-C data, which was then combined with 3D-DNA v180922 and rectified with Juicebox V1.11.08 (Durand et al. 2016).

Genome integrity assessment was performed using BUSCO v5.2.2 (Manni et al. 2021) based on the insecta_odb10 (*n* = 1,367) reference database. In addition, we mapped DNA- and RNA-sequencing reads to the assembled genome using Minimap2. SAMtools was used to count the mapping rate to check the raw data utilization and genome assembly integrity.

### Genome Annotation

The repeat-sequence database of *An. camellia* was created using the LTR search program (-LTRStruct) in RepeatModeler v2.0.2a based on ab initio prediction and repeat-sequence-specific structure, which was then merged with the RepBase-20181026 (Bao et al. 2015) and Dfam 3.3 (Hubley et al. 2016) databases. Finally, RepeatMasker v4.1.2pl (Flynn et al. 2020) was used to search for repeat sequences.

We used the MAKER v3.01.03 program to predict the structures of PCGs (Holt and Yandell 2011). We obtained two trained ab initio prediction tools GeneMark-ES/ET/EP 4.68_3.60_lic (Bruna et al. 2020) and Augustus v3.4 (Stanke et al. 2004) by using BRAKER v2.1.6 (Bruna et al. 2020). To improve prediction accuracy, transcriptomic and protein homology evidence was integrated. Using HISAT2 v2.2.0 (Kim et al. 2015), the transcriptome data were aligned to the genome. The OrthoDB10 v1 database was used to retrieve the protein sequences of arthropods (Kriventseva et al. 2019). Assembled and annotated genomes, protein sequences, and annotated GFF files of five closely related species (*Ap. mellifera*, *Nasonia vitripennis*, *Neodiprion lecontei*, *O. bicornis*, and *Solenopsis invicta*) were downloaded from NCBI. Gene prediction was performed using GeMoMa-1.8 (Keilwagen et al. 2019).

To annotate gene function, we adopted two strategies: gene functions were first predicted by comparison with the already existing database UniProtKB, and using the sensitive mode (–very-sensitive -e 1e-5) in Diamond v2.0.11.149 (Buchfink et al. 2015) to search UniProtKB for gene function. Furthermore, we compared the results with the corresponding databases: pfam (El-Gebali et al. 2019), Superfamily (Wilson et al. 2009), SMART (Letunic and Bork 2018), CDD (Marchler-Bauer et al. 2017), and eggNOG v5.0 (Huerta-Cepas et al. 2019) for the prediction of Reactome, KEGG pathways, and conserved sequences and domains in proteins. The first four and the last databases were obtained by searching through InterProScan 5.53-87 (Finn et al. 2017) and eggNOG-mapper v2.1.5 (Huerta-Cepas et al. 2017), respectively.

To annotate rRNA, snRNA, and miRNA, we compared them with well-known noncoding RNA libraries (Rfam database) by utilizing Infernal v1.1.4 (Nawrocki and Eddy 2013). Additionally, tRNA prediction was performed by utilizing tRNAscan-SE v2.0.9 (Chan and Lowe 2019).

### Gene Family Identification and Chromosomal Synteny

For gene family homology inference, protein-coding sequences of 10 Hymenoptera species were retrieved from NCBI (supplementary table S7, Supplementary Material online). These species included two Megachilidae species (*Anthidium xuezhong* and *O. bicornis*), two Apidae species (*Ap. mellifera* and *Bombus terrestris*), one Halictidae (*Dufourea novaeangliae*), one Pteromalidae (*N. vitripennis*), one Diprionidae (*N. lecontei*), one Formicidae (*S. invicta*), one Vespidae (*Vespa velutina*), and one Braconidae (*Aphidius gifuensis*). In order to find homologous gene families for each species, we used OrthoFinder v2.5.2 (Emms and Kelly 2019) for clustering protein sequences, and then protein sequences were compared by Diamond.

The genomes of *An. camellia*, *Ap. Mellifera*, and *O. bicornis* were assessed for chromosome-level gene content and shared gene order; we also compared *An. camellia* with *An. minutula*. Pairwise protein sequence analysis was performed using the Blastp in MMseq2 v12-113e3 with parameters of s 7.5 –alignment-mode 3 –num-iterations 4 -e 1e-5 –max-accept 5 (Steinegger and Soding 2017). In addition, this all.blast result file and the integrated comment file all.gff file were used as input files to obtain the covariance analysis results in MCScanX and visualized by TBtools v1.0692 (Chen et al. 2020).

## Supplementary Material

Supplementary materials are available at *Genome Biology and Evolution* online (http://www.gbe.oxfordjournals.org/).

## Supplementary Material

evad080_Supplementary_Data

## Data Availability

All raw sequencing and genome assembly data of *Andrena camellia* were deposited at NCBI under the accession number PRJNA839515.
