# Supplementary material for: A Chromosome-Level Genome Assembly and Evolution Analysis of Andrena camellia (Hymenoptera: Andrenidae)
Source: Genome Biol Evol. 2023 May 12;15(5):evad080. doi: 10.1093/gbe/evad080 (PMC10210616; doi:10.1093/gbe/evad080)
Supplement: evad080_Supplementary_Data [file evad080_supplementary_data.zip › Supplementary fig.pdf]

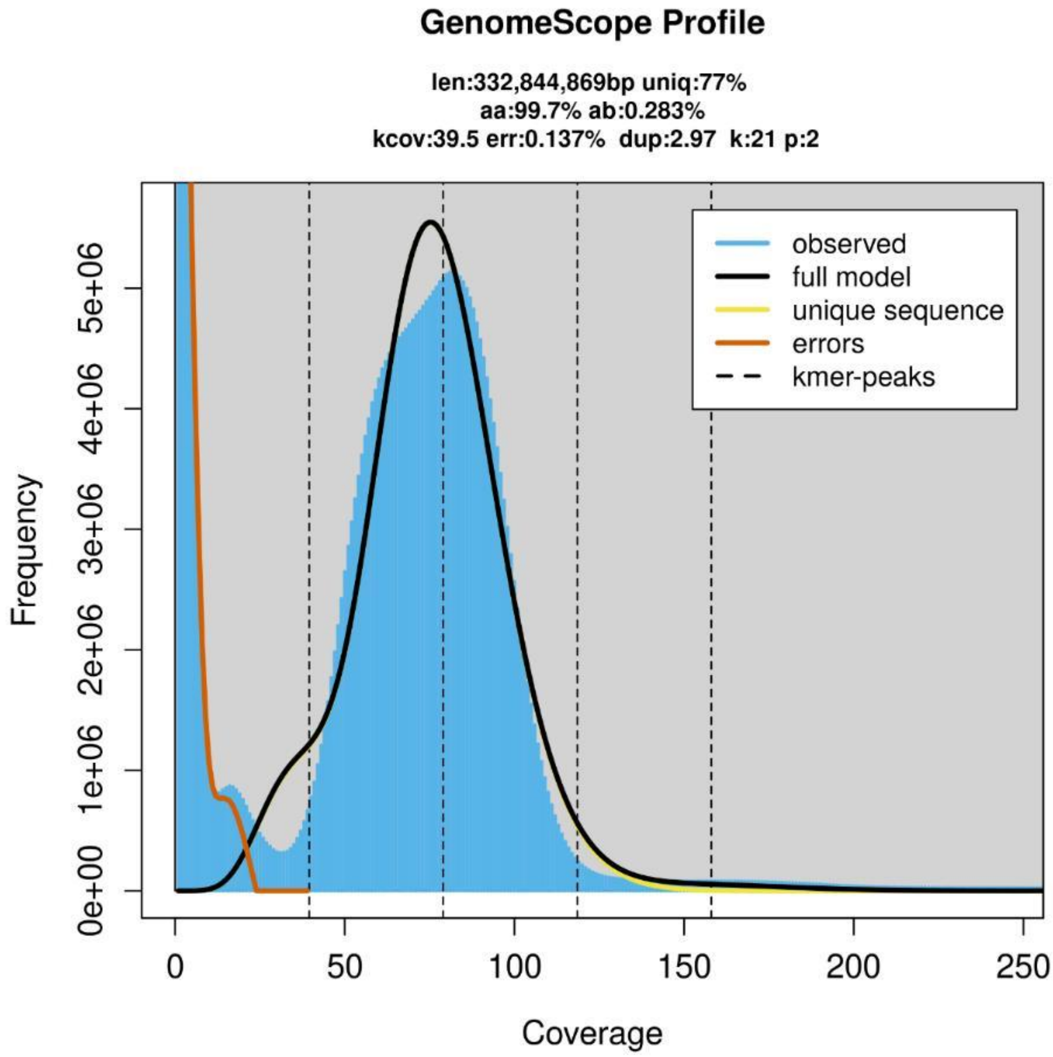

Fig. S1—GenomeScope profile plots of k-mer frequency at a k-mer length of 21 and a maximum k-mer coverage of 10,000.
